# The Role of PPARs in the Endothelium: Implications for Cancer Therapy

**DOI:** 10.1155/2008/904251

**Published:** 2008-11-24

**Authors:** David Bishop-Bailey, Karen E. Swales

**Affiliations:** Translational Medicine and Therapeutics, William Harvey Research Institute, Barts and The London School of Medicine and Dentistry, Queen Mary University London, Charterhouse Square, London EC1M 6BQ, UK

## Abstract

The growth and metastasis of cancers intimately involve the vasculature and in particular the endothelial cell layer. Tumours require new blood vessel formation via angiogenesis to support growth. In addition, inflammation, coagulation, and platelet activation are common signals in the growth and metastasis of tumour cells. The endothelium plays a central role in the homeostatic control of inflammatory cell recruitment, regulating platelet activation and coagulation pathways. PPAR*α*, -*β*/*δ*, and -*γ* are all expressed in endothelial cells. This review will discuss the roles of PPARs in endothelial cells in relation to angiogenesis, inflammation, coagulation, and platelet control pathways. In particular, we will discuss the recent evidence that supports the hypothesis that PPAR*α* and PPAR*γ* are antiangiogenic receptors, while PPAR*β*/*δ* is proangiogenic.

## 1. IMPORTANCE OF THE ENDOTHELIAL CELL IN CANCER

Endothelial cells play critical roles in vascular biology, being both the protective inner lining of vessels and the local site for delivery of oxygen to all tissues. It has become clear, particularly from the seminal work of Professor Judah Folkman, whom this issue is dedicated to, that the endothelium plays a critical role in the growth and spread of cancer [1–4]. The growth of tumours, or indeed any tissue growth requires new blood vessel formation to sustain it. This process of angiogenesis as a target for modulating cancer growth has been a major research theme. The critical initial stimulus for angiogenesis appears to be hypoxia in the growing tumour. The hypoxia leads to upregulation of hypoxia-induced transcription factors, for example, hypoxia inducible factor (HIF)-1*α* and HIF-2*α* [5–8], which stimulate the expressions of genes involved in oxygen homeostasis, and secretion of proangiogenic mediators such as vascular endothelial growth factor (VEGF) and basic fibroblast growth factor (bFGF) [4, 9, 10]. Although these are key growth factors for endothelial cell growth and morphogenesis, it is clear that there are an increasing number of endogenous proangiogenic factors (PGDF, IL-8, angiopoietin-1, leptin, matrix metalloproteinases, thrombin, plasminogen activators) and antiangiogenic factors (endostatin, angiostatin, thrombospondin-1, angiopoietin-2, IL-4, IL-12, IL-18, tissue inhibitor of MMPs, TGF-*β*, IFN*α*, -*β*, and -*γ*) [1, 4, 10, 11]. When the cumulative actions of the proangiogenic mediators outweigh their antiangiogenic counterparts an “angiogenic switch” occurs [12]. In particular, VEGF (VEGF-A; VEGF_165_) is a central mediator of endothelial cell growth and angiogenesis [13]. Two endothelial VEGF tyrosine kinase receptors have been identified: VEGFR-1/Flt-1, and VEGFR-2/KDR/Flk1, with the latter being the most important in VEGF-induced mitogenesis and permeability [13]. The lymphatic system and in particular lymphangiogenesis also contributes significantly to tumour metastasis. Unlike angiogenesis, where VEGF-(A) and VEGFR1/2 are key regulators, lymphangiogenesis is regulated by VEGFR-3 and VEGF-C/D isoforms (along with PROX1, podoplanin, LYVE-1, ephrinB2, and FOXC2) [14, 15]. Once stimulated by VEGF, the receptors initiate a signal transduction cascade, activating kinases such as ERK1/2 and Akt, which phosphorylate and activate further mediators of endothelial cell proliferation, apoptosis, and angiogenesis, such as eNOS [16].

The endothelium local to the tumour itself also contributes to tumour growth and metastasis via mechanisms independent of angiogenesis. Of increasing importance is the role of chronic inflammation in tumour progression. Chronic inflammation, in particular the presence of neutrophils, macrophages, and mast cells, correlates with poor prognosis and the angiogenic state of the tumour [17, 18]. The activation of the endothelium and its subsequent expression of adhesion molecules and chemokines is the interface for local inflammatory cell recruitment and extravasation. Central to these processes are proinflammatory transcription factors such as NF*κ*B. NF*κ*B regulates many inflammatory processes including inducible cytokine/chemokine and adhesion molecule expressions that are central to inflammatory cell recruitment, as well acting as a potent prosurvival signal within the cell [19].

In addition to angiogenesis and inflammation, cancer progression and metastasis is also facilitated by circulating cells and mediators regulated by the endothelium. The endothelium provides an antithrombotic surface and produces powerful antiplatelet and anticoagulant mediators such as prostacyclin, nitric oxide, and tissue- and urokinase-plasminogen activators [20]. Under physiological conditions, the endothelial surface is antithrombotic. Activated endothelial cells, however, are able to release prothrombotic/procoagulation mediators such as prostaglandin PGE_2_ [21, 22], plasminogen activator inhibitor (PAI)-1 [23], and tissue factor [23]. In cancer, thrombocytosis is common [24], suggesting that the physiological protective system usually provided by endothelial cells may be dysfunctional or overpowered by prothrombotic pathways. Driving this thrombosis may be tumour-derived thrombopoietin, and tumour- and platelet-derived growth factors and microparticles [24]. The consequence of activation of the coagulation cascade in cancer progression can be seen using thrombin as an example. Thrombin activates tumour cell adhesion to platelets and endothelial cells, and induces tumour cell growth, metastasis, and angiogenesis [25].

The movement of tumour cells into and out of the circulation (or the lymphatics) involves interaction with, and crossing of, the endothelial barrier. Although tumour endothelial cells are generally highly permeable (induced by factors such as VEGF), it is still unlikely that tumour cell movement is a passive process [26]. Within the circulation, transit of tumour cells is facilitated by their interactions with activated platelets [26]. The platelets are believed to act as a shield, protecting tumour cells from both physical forces and immune-mediated killing [26].

In summary, along with angiogenesis and lymphangiogenesis, endothelial cells regulate tumour progression not only by directly interacting with tumour cells, but also by regulating local inflammatory cell recruitment, the coagulation cascade, and platelet activity. When discussing the actions of PPARs in endothelial cells it is, therefore, important to consider all these properties.

## 2. PPARs AND ENDOTHELIAL CELLS

**Figure 1:**
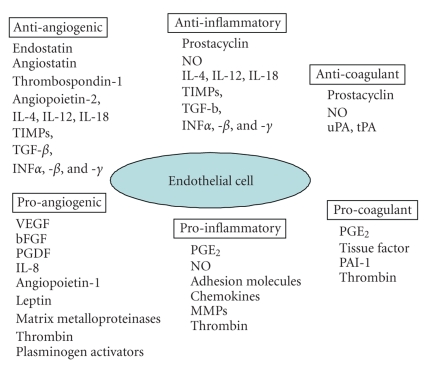
The endothelial cell is the interface between the circulation and underlying tissue, and as such plays an important homeostatic role both producing and responding to a variety of pro- and antiangiogenic, inflammatory, and coagulation factors. The balance between these opposing pathways is critical in the growth, development, spread, and metastasis of tumours.

PPAR*α*, PPAR*β*/*δ*, and PPAR*γ* are expressed in endothelial cells [27, 28], where they regulate cell proliferation, angiogenesis, inflammation, thrombosis, and coagulation (Figure 1). PPAR*α* is expressed in human aortic endothelial cells, carotid artery endothelial cells, and human umbilical vein endothelial cells [27, 29–31]. PPAR*γ* is similarly expressed in human endothelial cells both in vitro and in vivo [27, 28, 31, 32], while PPAR*β* is ubiquitously expressed. The role of PPAR*γ* has been well characterised in endothelial cell inflammation and angiogenesis [33, 34]. In contrast, the functions of PPAR*α* and PPAR*β*/*δ* in endothelial cells, especially in terms of angiogenesis, are only just beginning to be understood. Indeed, although the role of PPAR*γ* will be discussed in this review, since there is considerable information on PPAR*γ* in cancer [35] and an article on PPAR*γ* regulation of the angiogenic switch in this review series [36], this manuscript will focus more on recent observations highlighting novel roles for PPAR*α* and PPAR*β*/*δ* in endothelial cell function and in particular on the regulation of angiogenesis. The focus of this review is the endothelial cell, but it is important to note that PPAR*α*, *β*/*δ*, and *γ* expression and activity have been demonstrated in a variety of cancers, inflammatory cells [34], and in platelets [37–39]. Therefore, any effects of PPAR ligands on the development of cancer may be influenced by responses in these nonendothelial cell types as well.

## 3. PPAR*α* AND PPAR*γ*: ANTICANCER TARGETS IN THE ENDOTHELIUM

### 3.1. PPAR*α* and PPAR*γ* ligands

**Figure 2:**
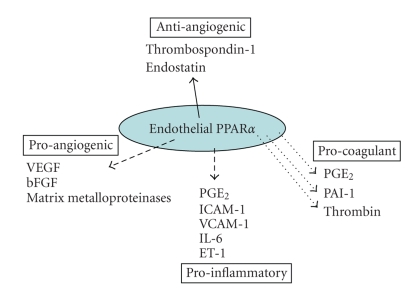
Endothelial PPAR*α* has predominantly inhibitory actions on endothelial cell activation. The majority of studies so far indicate that PPAR*α* activation induces (solid line) antiangiogenic factors, while reduces (broken line) proangiogenic factors, proinflammatory pathways, and procoagulant mediator release.

**Figure 3:**
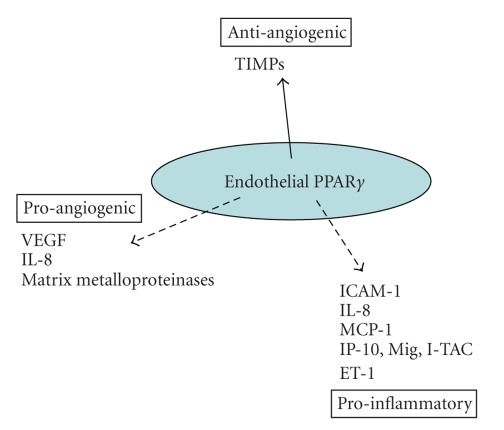
Endothelial PPAR*γ* has predominantly inhibitory actions on endothelial cell activation. The majority of studies so far indicate that PPAR*γ* activation inhibits (broken line) proangiogenic factors, proinflammatory pathways, and procoagulant mediator release, while inducing (solid line) antiangiogenic factors.

When discussing the roles of PPARs it is important to note the types of ligands potentially used in studies. Activators of PPAR*α* include a variety of eicosanoids, fatty acids, and synthetic compounds including the clinically used dyslipidemic drugs, the fibrates (gemfibrozil, fenofibrate, bezafibrate, ciprofibrate) [40, 41]. Similarly, PPAR*γ* activators also include a variety of eicosanoids, fatty acids, and synthetic compounds including the clinically used insulin sensitising thiazolidinedione drugs (rosiglitazone, pioglitizone, troglitizone (now withdrawn) [40, 41]. (See Figures 2 and 3.)

### 3.2. PPAR*α* and PPAR*γ* in cancer

One early observation regarding PPAR*α* activation by peroxisome proliferators was the induction of hepatocarcinogenesis in rodents; an effect absent in PPAR*α* (−/−) knockout mice [42, 43]. Although there has been a considerable amount of interest in the field, especially as the PPAR*α* activating fibrates are in clinical use, there is no evidence that long-term activation of PPAR*α* in nonrodent species including man is linked to hepatocarcinogenesis [42, 43].

In extrahepatic tissues, there have been fewer studies regarding PPAR*α* and cancer. Initially, it was suggested that PPAR*α* may prevent skin cancer [44, 45]. However, topical PPAR*α* agonists were only moderately protective against tumour promotion in mouse skin, despite the upregulation of PPAR*α* in tumours compared to normal epidermis [46]. Recent studies have revealed that PPAR*α* is commonly expressed in tumour cell lines, including lung, liver, leukaemia, prostate, pancreas, bladder, colon, glioblastoma, hemangioma, melanoma, ovarian, and breast [47–49]. PPAR*α* ligands inhibit the growth of colon, breast, endometrial, and skin cells in vitro [46, 48, 50–52] and human ovarian cancer [53], melanoma, lung carcinoma, glioblastoma, and fibrosarcoma [48]. PPAR*α* ligands also decrease tumour development in colon carcinogenesis [52] and inhibit melanoma cell metastasis in vitro and in vivo [50, 54].

PPAR*γ* is expressed in prostate, thyroid, colon, breast and hepatocellular carcinoma, gastric, pancreatic and lung cancer, neuroblastoma, astrocytoma, and glioma, where the receptors' ligands are antiproliferative and proapoptotic [35]. It is beyond the scope of this review to discuss all the findings of PPAR*γ* in cancer, and there are a number of excellent reviews in the field [33, 35, 55, 56] including one on PPAR*γ* and angiogenesis in this series [36].

The majority of the evidence points towards PPAR*γ* ligands suppressing tumourgenesis, for example, the receptors' ligands inhibit the growth of xenografts of many of the aforementioned tumours in vivo [35]. However, in colon cancer, the beneficial role for PPAR*γ* agonists is controversial [57]. In the APC^min^/+ mouse, PPAR*γ* ligands increased precancerous polyp formation and the frequency and size of tumours in the colon [58, 59]. In contrast, heterozygous loss of PPAR*γ* increases colon cancer incidence in mice [60]. This latter study corresponds with most of the available data, suggesting that PPAR*γ* has antineoplastic effects in colon cancer; a point further supported in colon cancer patient studies by the detection of mutations causing loss of function or impaired ligand binding of PPAR*γ* [61] and polymorphisms of the PPAR*γ* gene [62].

There have been positive results using PPAR*γ* ligands to treat tumours experimentally both in vitro and in vivo, but so far this has not been successfully translated into a beneficial anticancer therapy in man. There have been a number of small scale clinical trials testing PPAR*γ* ligands in cancer in man with varying success [63]. The most promising results were from small phase II studies treating prostate cancer [64] and liposarcoma patients [65] with troglitazone. In contrast, a phase II study treating liposarcoma patients with rosiglitazone did not significantly improve clinical outcome [66] and so far no beneficial effects of PPAR*γ* ligands have been observed in trials for breast or colon cancer patients [35].

### 3.3. PPAR*α* and PPAR*γ* regulation of angiogenesis

Early studies showed no effect of the selective PPAR*α* ligand WY-14643 on endothelial cell proliferation [27], however, recent studies using immortalised human dermal microvascular endothelial cells show that the PPAR*α* ligand fenofibrate inhibits endothelial cell proliferation, migration, and tube formation (on a fibrin matrix) in vitro and angiogenesis in vivo [67]. Fenofibrate acts by disrupting the formation of the actin cytoskeleton and inhibits bFGF-induced Akt activation and cyclooxygenase 2 (COX-2) gene expression [67]. Similar results were found in a porcine model of vascular remodelling after coronary artery angioplasty where fenofibrate increased lumen size and vessel area and inhibited constrictive remodelling and inflammatory cell infiltration [68]. Importantly, adventitial angiogenesis was significantly reduced by fenofibrate in the injured vessels 3 days after angioplasty [68].

In contrast to this vascular study, the investigation of PPAR*α* regulation of tumour angiogenesis has only just begun. In a recent report, Panigraphy et al. provide compelling evidence for PPAR*α* inhibition of tumour growth by targeting angiogenesis [48]. Similar to previous findings, PPAR*α* activation had direct effects on endothelial cells, inhibiting VEGF-induced endothelial cell migration in vitro and FGF2 induced corneal angiogenesis in vivo [48]. Tumour cell synthesis of VEGF and FGF2 was also suppressed by PPAR*α* activation in conjunction with an increased expression of antiangiogenic thrombospondin-1 (TSP-1) [48]. In subcutaneously implanted human pancreatic cancer cells grown in mice, as well as in human prostate cancer, PPAR*α* expression was detected not only in the tumour cells, but also in the new invading microvessels [48]. Systemic treatment of mice with PPAR*α* ligands inhibited the growth of melanoma, glioblastoma, and fibrosarcoma tumours implanted in vivo, which was associated with a reduction in vessel density and inflammation [48]. To dissect the mechanism by which PPAR*α* suppressed tumour growth (i.e., direct effects on the tumour and/or angiogenesis), embryonic fibroblasts from PPAR*α* (−/−) knockout mice were transformed with SV40 large T antigen and H-ras oncogenes then implanted into wild-type and PPAR*α*−/− mice. The growth of these cells into tumours could be suppressed by PPAR*α* ligands in wild-type mice only, indicating that tumour suppression by PPAR*α* ligands was completely dependent on the expression of PPAR*α* in the host but not in the tumour cells [48]. Fenofibrate strongly induced the antiangiogenic factors TSP-1 and endostatin in wild-type, but not PPAR*α*−/− mice, supporting the role of PPAR*α* as an antiangiogenic regulator [48]. Angiogenesis and inflammation are central processes through which the tumour interacts with its surroundings to influence tumour growth. Although this study does not rule out an anti-inflammatory effect of the PPAR*α* ligands, it is highly unlikely that the antitumour host-derived effects are due to suppression of inflammation because mice deficient in PPAR*α* generally exhibit enhanced inflammation [64].

TSP-1 is a potent angiogenesis inhibitor that targets endothelial cells for apoptosis by initiating a signalling cascade through the CD36 receptor. PPAR*α* directly induces TSP-1 and can enhance TSP-1 signalling indirectly by upregulating CD36 in the endothelium. PPAR*α* activation upregulates CD36 expression in the liver [69] and in macrophages [70]. Moreover, coadministration of PPAR*γ* ligands with exogenous TSP-1 or the TSP-1 peptide derivative ABT510 synergises to suppress angiogenesis and induce endothelial cell apoptosis [71]. The improvement of the antiangiogenic efficacy of TSP-1 was attributed to PPAR*γ*-induced CD36 expression via a PPAR response element in the CD36 promoter [69, 71].

The vast majority of studies have indicated an antiangiogenic role for PPAR*α* and PPAR*γ* in a variety of models. However, it is important to note that the VEGF promoter contains a PPAR response element and PPAR*α* and -*γ* ligands can induce VEGF in certain cell types [72–75]. Moreover, in contrast to the majority of findings, a recent study suggests that both PPAR*α* and PPAR*γ* ligands may also have proangiogenic properties in vitro in an endothelial/interstitial cell coculture assay and in a murine corneal angiogenesis model in vivo [72]. The angiogenesis induced by PPAR*α* and PPAR*γ* ligands was associated with the induction of VEGF, accompanied by increased activation of AKT and eNOS (by phosphorylation) [72]. How the levels of PPAR*α*- or PPAR*γ*-mediated angiogenesis are compared to traditional growth factor-induced angiogenesis is not known? Indeed, these results are controversial, as previous corneal angiogenesis models clearly demonstrate antiangiogenic effects of PPAR*α* and PPAR*γ* ligands [28, 48, 76].

Multiple mechanisms have been proposed by which PPAR*α* and PPAR*γ* regulate the changes in pro- and antiangiogenic factors. Here, we will focus on the central target for PPAR regulation of angiogenesis, the proangiogenic VEGF/VEGFR signalling pathway. PPAR*γ* can downregulate VEGF either directly through a PPAR response element within the VEGF promoter [77] or by decreasing PGE_2_, an endogenous stimulator of angiogenesis [78]. PPAR*γ* can also decrease VEGF responses by suppressing transcription of its receptor VEGFR2, by interacting with and preventing Sp1 binding to DNA [79].

In colorectal cancer cell lines, PPAR*α* also inhibits the transcription factor AP-1, impairing its binding to response elements in the VEGF and COX-2 genes and inhibiting c-jun transactivation activity, thus downregulating VEGF and COX-2 expression [80]. It is, therefore, clear that the regulation of angiogenic factors by PPAR*α* and PPAR*γ* may be determined by cell and cancer type and the experimental models used. Much more research is required to fully understand whether PPAR activation will be pro- or antiangiogenic in specific human cancers.

### 3.4. The effects of PPAR*α* and PPAR*γ* on endothelial progenitor cells

Endothelial progenitor cells (EPCs) present in peripheral blood promote angiogenesis and improve endothelial function. The research on the effects of PPARs on EPCs has focused on PPAR*γ*. Despite PPAR*γ* generally being considered antiangiogenic, the PPAR*γ* ligands rosiglitazone and pioglitazone in diabetic patients increase endothelial progenitor cell (EPC) number and migratory activity [81, 82]. Pioglitazone and rosiglitazone also improve the adhesive capacity of EPCs to fibronectin and collagen [82] and promote EPC colony formation, [83, 84]. In vitro, pioglitazone increased EPC proliferation, colony formation, and attenuated apoptosis [85]. Similarly, in mice pioglitazone induced the number and migratory activity of EPCs while decreasing their apoptosis, resulting in increased in vivo neoangiogenesis [86]. From these results, it has been proposed that PPAR*γ* ligands may have a double-edged role in angiogenesis, with proangiogenic effects on EPCs at low-systemic concentrations and antiangiogenic effects at higher local concentrations [86]. Indeed, biphasic effects of pioglitazone were observed on EPCs in culture, when the number of EPC colonies and amount of adhesion were increased by 1 *μ*M but not 10 *μ*M [87]. This higher concentration of pioglitazone induced TGF-*β*1 and its receptor endogolin, which suppress EPC function [87]. These findings have important clinical implications suggesting that the pro-/antiangiogenic properties of PPAR*γ* ligands may be largely dose-driven. Moreover, understanding this mechanism by which PPAR*γ* may regulate both pro- and antiangiogenic pathways at least in EPCs may help to explain some of the contradictions in the studies examining the role of PPAR*γ* in angiogenesis.

### 3.5. Effects of PPAR*α* and PPAR*γ* on endothelial cell inflammation

The role of PPAR*α* in inflammation has been studied in animal models, particularly in wound healing and cardiovascular disease models (atherosclerosis and restenosis) [55, 56]. PPAR*α* is a negative regulator of inflammation [34] in inflammatory models. Supporting this, PPAR*α*−/− mice exhibit enhanced inflammation [88], although this may be due in part to deceased *β*-oxidation and accumulation of biologically active lipid mediators.

In addition to these experimental models, PPAR*α* agonists decrease the expression of inflammatory markers both in human cells and patients treated with fibrates [89, 90]. In human endothelial cells in culture, PPAR*α* ligands inhibit the cytokine/LPS induction of COX-2 [38, 69], ICAM-1 [91], VCAM-1 [29, 31], endothelin-1 [92], IL-6, and prostaglandin E_2_ [32, 93]. Similarly, PPAR*α* ligands repress thrombin-induced expression of endothelin-1 [32]. The PPAR*α* ligand fenofibrate, but not the PPAR*γ* ligand rosiglitazone, also reduces the induction of tissue factor in human endothelial cells [94], while PAI-1 levels remain unchanged [31]. PPAR*α* inhibits proinflammatory mediators by interfering with the transactivation activity of NF*κ*B and AP-1, the main transcription factors mediating inflammatory and growth factor responses. PPAR*α* via direct protein-protein interactions can bind and inhibit the actions p65 and c-jun subunits, respectively [95, 96].

Although the weight of evidence points towards an anti-inflammatory role for PPAR*α*, oxidised lipids that can activate PPAR*α* have been shown to increase the release of neutrophil chemoattractant IL-8 and MCP-1 from endothelial cells [30]. Similarly, PPAR*α* ligands induce COX-2 in human breast and colon cancer cells [97, 98].

PPAR*γ*, similarly, is a well-established negative regulator of the inflammatory response in vitro and in vivo [34]. PPAR*γ* agonists have been shown to mediate effects on cell survival, surface-protein expression, and cytokine and chemokine production. In endothelial cells, PPAR*γ* ligands can induce apoptosis [27] and decrease inflammatory cell recruitment by inhibiting the production of chemokines IL-8, MCP-1 [30, 99], IP-10, Mig, and I-TAC [100] and reducing ICAM-1 expression [101]. Similar to PPAR-*α*, PPAR*γ* ligands repress thrombin-induced expression of endothelin-1 [32].

## 4. PPAR*β*/*δ*

### 4.1. PPAR*β*/*δ* ligands

**Figure 4:**
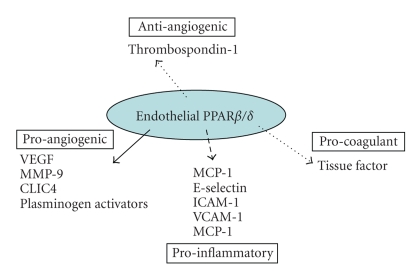
Endothelial PPAR*β*/*δ* has predominantly proangiogenic actions on endothelial cells. The majority of studies so far indicate that PPAR*β*/*δ* activation induces (solid line) proangiogenic factors, while reduces (broken line) antiangiogenic factors. Similar to PPAR*α* and PPAR*γ*, PPAR*β*/*δ* also appears to be anti-inflammatory by reducing proinflammatory pathways and potentially anticoagulant by reducing tissue factor release.

PPAR*β*/*δ* (Figure 4) is almost ubiquitously expressed [102], although compared to PPAR*α* and -*γ*, less is known regarding its role in the body. However, like PPAR*α* and -*γ*, it appears able to regulate lipid metabolism, cellular proliferation, and the inflammatory response [55, 56]. Activators of PPAR*β*/*δ* include a variety of eicosanoids (the COX product prostacyclin [40, 41], COX/prostacyclin synthase-derived endocannabinoid metabolites [103]); fatty acids and synthetic compounds including GW0742X, GW501516, L-165,461, and compound F [40, 41].

### 4.2. PPAR*β*/*δ* and cancer

There has recently been an increasing amount of contradictory literature published regarding PPAR*β*/*δ* regulation of tumour cell growth and tumour cell release of VEGF. PPAR*β*/*δ* ligands induce VEGF in bladder cancer [104], human breast (T47D, MCF7) and prostate (LNCaP, PNT1A) cancer cell lines, along with its receptor VEGFR1 [105], but not in colon (HT29, HCT116, LS-174T) and hepatoma (HepG2, HuH7) cell lines [106].

Much of the research into PPAR*β*/*δ* in cancer has focused on gastrointestinal cancer. PPAR*β*/*δ* expression is enhanced in human and rodent colorectal tumours, as well as preneoplastic colonic mucosa [107, 108]. PPAR*β*/*δ* is transcriptionally regulated by *β*-catenin/Tcf-4, which can be suppressed APC. Therefore, in colorectal cancer cells that commonly carry an APC mutation, PPAR*β*/*δ* is upregulated [108]. Interestingly, PPAR*β*/*δ* accumulation was localised to human colorectal carcinoma cells with a highly malignant morphology [109], suggesting PPAR*β*/*δ* promotes tumourogenesis. Supporting this theory, the growth of PPAR*β*/*δ*−/− HCT-116 human colon carcinoma cell xenografts was reduced compared to wild-type PPAR*β*/*δ* expressing cells [83].

Using animal models, a positive link has been made between PPAR*β*/*δ* and colon cancer development, especially using the intestinal polyp model, APC^min^/+ mice. In this model, deletion of PPAR*β*/*δ* decreases intestinal adenoma growth and inhibits the tumour-promoting effects of the PPAR*β*/*δ* agonist GW501516 [85, 110]. PPAR*β*/*δ* activation induces VEGF in colon carcinoma cells, promoting cell survival by activation of Akt signalling [85]. Angiogenesis was not studied in these experiments, however, for a tumour to grow greater than 2 mm in diameter a functional vessel network is required [111]. Indeed, the most prominent effect of PPAR*β*/*δ* activation in APC^min^/+ mice, observed by Gupta et al., was a significant increase in the number of polyps greater than 2 mm in diameter [110]. Whereas there was a significant decrease in the growth of polyps greater than 2 mm in diameter in PPAR*β*/*δ*−/− APC^min^/+ mice, despite a lack of effect on overall polyp incidence [112]; indicating that PPAR*β*/*δ* promotes tumour growth via angiogenesis.

In contrast, deletion of PPAR*β*/*δ* in APC^min^/+ mice enhanced colon polyp formation in untreated mice and in mice with chemically induced colon carcinogenesis [113, 114]. The PPAR*β*/*δ* ligand GW0742 inhibited chemically induced colon carcinogenesis in PPAR*β*/*δ* wild-type but not PPAR*β*/*δ*−/− mice [115]. The differences between these contrasting results have been suggested to be due to differences in genetic background, breeding, or the PPAR*β*/*δ* knockout strategy of the APC^min^/+ mouse models [116]. However, this would not explain why in human colon and liver cancer cell lines, PPAR*β*/*δ* ligands had no effect on cell growth, Akt phosphorylation, or VEGF and COX-2 expression in vitro or on these markers in the liver, colon and colon polyps in mice treated in vivo [106]. The role of PPAR*β*/*δ* in VEGF-mediated tumourgenesis, therefore, still requires further study and clarification.

### 4.3. PPAR*β*/*δ* and angiogenesis

Initial reports using prostacyclin as a ligand suggested that similar to PPAR*α* and PPAR*γ*, PPAR*β*/*δ* promoted endothelial cell apoptosis [117], and potentially decreased angiogenesis. In contrast, with the development of highly selective synthetic ligands, there is an increasing evidence to propose a role for PPAR*β*/*δ* in regulating endothelial cell survival, proliferation, and angiogenesis. Indeed, treating endothelial cells with the selective PPAR*β*/*δ* ligand GW501516 induces proliferation, VEGF receptor (Flt-1; VEGF R1) expression, and VEGF production [105, 118]. In addition to inducing proliferation, PPAR*β*/*δ* also protects the endothelial cell from oxidant injury via induction of the antiapoptotic and anti-inflammatory protein 14-3-3*α* [119].

PPAR*β*/*δ* potently induces angiogenesis by human and murine vascular endothelial cells in tumour extracellular matrix in vitro and in a murine matrigel plug model in vivo [118]. The stimulated release of VEGF from human endothelial cells was a major trigger for morphogenesis, although mRNA for the matrix metalloproteinase (MMP)-9, a protease important for cell migration, was also elevated [118]. In addition to VEGF, genomic and proteomic analysis of PPAR*β*/*δ*−/− endothelial cells isolated from matrigel plugs identified a number of additional candidate genes that may mediate the angiogenic actions of PPAR*β*/*δ*. Cdkn1c, which encodes the cell cycle inhibitor p57^Kip2^, is induced by PPAR*β*/*δ* [120]. The chloride intracellular channel protein (CLIC)-4 is decreased in migrating endothelial cells from PPAR*β*/*δ* knockout mice, whereas the expression of cellular retinol binding protein CRBP1 is increased [121]. CLIC-4 plays an essential role during tubular morphogenesis [122], while CRBP1 inhibits cell survival pathways by blocking the Akt signalling pathway [123]. The combination of these studies indicates that PPAR*β*/*δ* may induce endothelial cell mitogenesis and differentiation signals, including VEGF, 14-3-3*α*, CLIC4, CRBP-1, and p57^KIP2^, which may combine to bring about the functional morphogenic changes associated with the angiogenic switch.

Two recent studies in particular have addressed the regulation of angiogenesis by PPAR*β*/*δ* in matrigel plugs in PPAR*β*/*δ* wild-type and knockout mice [120, 124]. Xenograft tumours in PPAR*β*/*δ*−/− mice exhibited a diminished blood flow and immature hyperplastic microvascular structures when compared to wild-type mice. Moreover, the reintroduction of PPAR*β*/*δ* into the matrigel plugs was able to rescue the knockout phenotype by triggering microvessel maturation [120]. In addition, tumour angiogenesis and growth are markedly inhibited in PPAR*β*/*δ*−/− mouse models of subcutaneous Lewis lung carcinoma and B16 melanoma. PPAR*β*/*δ* expression correlated with advanced pathological tumour stage and increased risk for tumour recurrence and distant metastasis in pancreatic tumours from patients who had undergone the “angiogenic switch” [124]. PPAR*β*/*δ* has, therefore, been suggested as a “hub node” transcription factor, regulating the tumour angiogenic switch [124].

### 4.4. The effects of PPAR*γ* *β*/*δ* on endothelial progenitor cells

Little is known about the effects of PPAR*β*/*δ* on EPCs, but there is one study that shows that PPAR*β*/*δ* is a key regulator of EPC proangiogenic functions. Prostacyclin is a putative PPAR*β*/*δ* ligand and proangiogenic factor, produced by COX and PGI_2_ synthase in the endothelium. EPC tube formation and proliferation are induced by the selective PPAR*β*/*δ* ligand GW510516. EPCs treated with an inhibitor of COX or COX-1, prostacyclin synthase, or PPAR*β*/*δ* specific siRNA, exhibit decreased cell proliferation and tube formation [125]. Thus the proangiogenic effects of human EPCs appear in part dependent on the biosynthesis of prostacyclin and the subsequent activation of PPAR*β*/*δ*.

### 4.5. The effect of PPAR*β*/*δ* on endothelial cell inflammation

Little is known regarding the role of PPAR*β*/*δ* in endothelial cell inflammation and mediator secretion. PPAR*β*/*δ* ligands, similar to PPAR*α* and PPAR*γ* ligands, inhibit cytokine-stimulated upregulation of adhesion molecules ICAM-1, VCAM-1, and e-selectin and NF*κ*B translocation [126, 127]. These anti-inflammatory effects of PPAR*β*/*δ* in endothelial cells occur when the complex between PPAR*β*/*δ* and the transcriptional repressor BCL6 is removed by ligand activation, identical to the mechanism identified in monocytes [128]. PPAR*β*/*δ* and BCL6 are then free to act on PPAR*β*/*δ* targets (including SOD and catalase) and BCL6 targets which importantly include the repression of NF*κ*B. In addition to anti-inflammatory effects, endogenous PPAR*β*/*δ* ligands are continuously produced in endothelial cells to suppress the release of tissue factor, the primary initiator of coagulation [103].

## 5. PPAR THERAPY FOR CANCER

The PPARs have pleiotrophic actions on nonvascular and vascular cells. PPAR*α* and PPAR*γ* ligands (although there are well-detailed current concerns for rosiglitazone) are in clinical use, are considered safe, and have high tolerability with chronic use. There is considerable evidence that PPAR*γ* and increasing evidence that PPAR*α* are vascular protective and reduce angiogenesis. Unfortunately, as yet, there is a little clinical evidence to support these actions, apart from the promising results with the PPAR*γ* ligand troglitazone in liposarcoma and prostate cancer previously mentioned [64, 65]. Clinically, PPAR*α* and *γ* ligands do not appear to be strong antiangiogenic drugs. However, since PPAR*α* and PPAR*γ* ligands are in clinical use and lack severe side effects, the potential for their use to complement or augment current and new therapies to treat a variety of cancers is currently being tested in small scale trials. For example, a phase II trial combining anti-inflammatory and angiostatic therapy (PPAR*γ* ligand pioglitazone and COX-2 inhibitor, rofecoxib) with metronomic low-dose chemotherapy (trofosamide) found that the progression-free survival rates of advanced melanoma patients were longer with the combination treatment than with metronomic chemotherapy alone [129]. This combination therapy was also successful in achieving disease stabilization or remission in patients with advanced progressive malignant vascular tumours [130] and partial remission in a single patient with endemic Kaposi sarcoma [131]. However, a similar phase II study on high-grade glioma patients, showed disease stabilisation in only 4 out of 14 patients, suggesting that this combined therapy may only be suitable for a subset of patients [132]. The COX-2 inhibitor rofecoxib was included in the trial because COX-2 plays a role in endothelial tube formation, pericyte recruitment, and endothelial cell survival during early angiogenesis [133]. As PPAR*α* and *γ* ligands have been shown to inhibit COX-2 induction in endothelial cells, it would be interesting to test the combined effects of PPAR*α* or −*γ* ligands with metronomic chemotherapy alone.

In contrast to PPAR*α* and PPAR*γ*, there is increasing evidence that PPAR*β*/*δ* is proangiogenic and an important transcription factor in the angiogenic switch. PPAR*β*/*δ* has an interesting activity profile in that like the other PPARs it also appears to have anti-inflammatory properties. As PPAR*β*/*δ* is considered a target to treat dyslipidaemia, its proangiogenic properties should, therefore, be considered in the long-term use of PPAR*β*/*δ* ligands to treat chronic metabolic diseases. The development of selective antagonists for PPAR*β*/*δ* offers great potential for cancer treatment. One such antagonist has recently been identified, GSK0660, which can compete with agonist in a cellular context and by itself exhibits inverse agonist activity [134]. This antagonist appears to act by promoting PPAR*β*/*δ*-mediated repression of gene expression. Unfortunately, this compound lacks in vivo bioavailability, but will be a valuable tool for elucidating the role of PPAR*β*/*δ* in cancer and angiogenesis in vitro and a basis for further development of a selective bioavailable PPAR*β*/*δ* antagonist [134]. Selective modulators of PPAR*β*/*δ*, which maintain the beneficial metabolic (and anti-inflammatory) effects while exerting no proangiogenic effects would also be beneficial. Interestingly, there is a newly developed PPAR-*α* agonist (R)-K-13675, which inhibits the secretion of inflammatory markers without affecting cell proliferation or endothelial tube formation [135], which suggests that selective modulators for the other PPARs may soon be available.

## References

[1] Folkman J (2002). Role of angiogenesis in tumor growth and metastasis. *Seminars in Oncology*.

[2] Folkman J (2003). Fundamental concepts of the angiogenic process. *Current Molecular Medicine*.

[3] Folkman J (2003). Angiogenesis and apoptosis. *Seminars in Cancer Biology*.

[4] Folkman J (2006). Angiogenesis. *Annual Review of Medicine*.

[5] Blancher C, Moore JW, Talks KL, Houlbrook S, Harris AL (2000). Relationship of hypoxia-inducible factor (HIF)-1*α* and HIF-2*α* expression to vascular endothelial growth factor induction and hypoxia survival in human breast cancer cell lines. *Cancer Research*.

[6] Marti HH (2005). Angiogenesis—a self-adapting principle in hypoxia. *EXS*.

[7] Maxwell PH, Ratcliffe PJ (2002). Oxygen sensors and angiogenesis. *Seminars in Cell & Developmental Biology*.

[8] Wang GL, Semenza GL (1993). General involvement of hypoxia-inducible factor 1 in transcriptional response to hypoxia. *Proceedings of the National Academy of Sciences of the United States of America*.

[9] Presta M, Dell'Era P, Mitola S, Moroni E, Ronca R, Rusnati M (2005). Fibroblast growth factor/fibroblast growth factor receptor system in angiogenesis. *Cytokine & Growth Factor Reviews*.

[10] Ribatti D, Conconi MT, Nussdorfer GG (2007). Nonclassic endogenous novel regulators of angiogenesis. *Pharmacological Reviews*.

[11] Folkman J (2006). Antiangiogenesis in cancer therapy—endostatin and its mechanisms of action. *Experimental Cell Research*.

[12] Naumov GN, Bender E, Zurakowski D (2006). A model of human tumor dormancy: an angiogenic switch from the nonangiogenic phenotype. *Journal of the National Cancer Institute*.

[13] Hoeben A, Landuyt B, Highley MS, Wildiers H, Van Oosterom AT, De Bruijn EA (2004). Vascular endothelial growth factor and angiogenesis. *Pharmacological Reviews*.

[14] Chang L, Kaipainen A, Folkman J (2002). Lymphangiogenesis: new mechanisms. *Annals of the New York Academy of Sciences*.

[15] Wissmann C, Detmar M (2006). Pathways targeting tumor lymphangiogenesis. *Clinical Cancer Research*.

[16] Ying L, Hofseth LJ (2007). An emerging role for endothelial nitric oxide synthase in chronic inflammation and cancer. *Cancer Research*.

[17] de Visser KE, Eichten A, Coussens LM (2006). Paradoxical roles of the immune system during cancer development. *Nature Reviews Cancer*.

[18] Lin EY, Pollard JW (2004). Role of infiltrated leucocytes in tumour growth and spread. *British Journal of Cancer*.

[19] Karin M (2006). Nuclear factor-*κ*B in cancer development and progression. *Nature*.

[20] van Hinsbergh VWM (2001). The endothelium: vascular control of haemostasis. *European Journal of Obstetrics Gynecology and Reproductive Biology*.

[21] Gross S, Tilly P, Hentsch D, Vonesch J-L, Fabre J-E (2007). Vascular wall-produced prostaglandin E2 exacerbates arterial thrombosis and atherothrombosis through platelet EP3 receptors. *Journal of Experimental Medicine*.

[22] Hla T, Neilson K (1992). Human cyclooxygenase-2 cDNA. *Proceedings of the National Academy of Sciences of the United States of America*.

[23] Feinbloom D, Bauer KA (2005). Assessment of hemostatic risk factors in predicting arterial thrombotic events. *Arteriosclerosis, Thrombosis, and Vascular Biology*.

[24] Sierko E, Wojtukiewicz MZ (2007). Inhibition of platelet function: does it offer a chance of better cancer progression control?. *Seminars in Thrombosis and Hemostasis*.

[25] Nierodzik ML, Karpatkin S (2006). Thrombin induces tumor growth, metastasis, and angiogenesis: evidence for a thrombin-regulated dormant tumor phenotype. *Cancer Cell*.

[26] Gupta GP, Massagué J (2006). Cancer metastasis: building a framework. *Cell*.

[27] Bishop-Bailey D, Hla T (1999). Endothelial cell apoptosis induced by the peroxisome proliferator-activated receptor (PPAR) ligand 15-deoxy-Δ^12,14^-prostaglandin J_2_. *The Journal of Biological Chemistry*.

[28] Xin X, Yang S, Kowalski J, Gerritsen ME (1999). Peroxisome proliferator-activated receptor *γ* ligands are potent inhibitors of angiogenesis in vitro and in vivo. *The Journal of Biological Chemistry*.

[29] Jackson SM, Parhami F, Xi X-P (1999). Peroxisome proliferator-activated receptor activators target human endothelial cells to inhibit leukocyte-endothelial cell interaction. *Arteriosclerosis, Thrombosis, and Vascular Biology*.

[30] Lee H, Shi W, Tontonoz P (2000). Role for peroxisome proliferator-activated receptor *α* in oxidized phospholipid-induced synthesis of monocyte chemotactic protein-1 interleukin-8 by endothelial cells. *Circulation Research*.

[31] Marx N, Bourcier T, Sukhova GK, Libby P, Plutzky J (1999). PPAR*γ* activation in human endothelial cells increases plasminogen activator inhibitor type-1 expression: PPAR*γ* as a potential mediator in vascular disease. *Arteriosclerosis, Thrombosis, and Vascular Biology*.

[32] Delerive P, Martin-Nizard F, Chinetti G (1999). Peroxisome proliferator-activated receptor activators inhibit thrombin-induced endothelin-1 production in human vascular endothelial cells by inhibiting the activator protein-1 signaling pathway. *Circulation Research*.

[33] Giaginis C, Margeli A, Theocharis S (2007). Peroxisome proliferator-activated receptor-*γ* ligands as investigational modulators of angiogenesis. *Expert Opinion on Investigational Drugs*.

[34] Moraes LA, Piqueras L, Bishop-Bailey D (2006). Peroxisome proliferator-activated receptors and inflammation. *Pharmacology & Therapeutics*.

[35] Grommes C, Landreth GE, Heneka MT (2004). Antineoplastic effects of peroxisome proliferator-activated receptor *γ* agonists. *Lancet Oncology*.

[36] Giaginis C, Tsantili-Kakoulidou A, Theocharis S (2008). Peroxisome proliferator-activated receptor-*γ* ligands: potential pharmacological agents for targeting the angiogenesis signaling cascade in cancer. *PPAR Research*.

[37] Akbiyik F, Ray DM, Gettings KF, Blumberg N, Francis CW, Phipps RP (2004). Human bone marrow megakaryocytes and platelets express PPAR*γ*, and PPAR*γ* agonists blunt platelet release of CD40 ligand and thromboxanes. *Blood*.

[38] Ali FY, Davidson SJ, Moraes LA (2006). Role of nuclear receptor signaling in platelets: antithrombotic effects of PPAR*β*. *The FASEB Journal*.

[39] Moraes LA, Swales KE, Wray JA (2007). Nongenomic signaling of the retinoid X receptor through binding and inhibiting Gq in human platelets. *Blood*.

[40] Bishop-Bailey D (2000). Peroxisome proliferator-activated receptors in the cardiovascular system. *British Journal of Pharmacology*.

[41] Bishop-Bailey D, Wray J (2003). Peroxisome proliferator-activated receptors: a critical review on endogenous pathways for ligand generation. *Prostaglandins and Other Lipid Mediators*.

[42] Gonzalez FJ, Shah YM (2008). PPAR*α*: mechanism of species differences and hepatocarcinogenesis of peroxisome proliferators. *Toxicology*.

[43] Peters JM, Cheung C, Gonzalez FJ (2005). Peroxisome proliferator-activated receptor-*α* and liver cancer: where do we stand?. *Journal of Molecular Medicine*.

[44] Hanley K, Jiang Y, He SS (1998). Keratinocyte differentiation is stimulated by activators of the nuclear hormone receptor PPAR*α*. *Journal of Investigative Dermatology*.

[45] Kömüves LG, Hanley K, Man M-Q, Elias PM, Williams ML, Feingold KR (2000). Keratinocyte differentiation in hyperproliferative epidermis: topical application of PPAR*α* activators restores tissue. *Journal of Investigative Dermatology*.

[46] Thuillier P, Anchiraico GJ, Nickel KP (2000). Activators of peroxisome proliferator-activated receptor-*α* partially inhibit mouse skin tumor promotion. *Molecular Carcinogenesis*.

[47] Collett GP, Betts AM, Johnson MI (2000). Peroxisome proliferator-activated receptor *α* is an androgen-responsive gene in human prostate and is highly expressed in prostatic adenocarcinoma. *Clinical Cancer Research*.

[48] Panigrahy D, Kaipainen A, Huang S (2008). PPAR*α* agonist fenofibrate suppresses tumor growth through direct and indirect angiogenesis inhibition. *Proceedings of the National Academy of Sciences of the United States of America*.

[49] Suchanek KM, May FJ, Robinson JA (2002). Peroxisome proliferator-activated receptor *α* in the human breast cancer cell lines MCF-7 and MDA-MB-231. *Molecular Carcinogenesis*.

[50] Grabacka M, Placha W, Plonka PM (2004). Inhibition of melanoma metastases by fenofibrate. *Archives of Dermatological Research*.

[51] Saidi SA, Holland CM, Charnock-Jones DS, Smith SK (2006). In vitro and in vivo effects of the PPAR-alpha agonists fenofibrate and retinoic acid in endometrial cancer. *Molecular Cancer*.

[52] Tanaka T, Kohno H, Yoshitani S-I (2001). Ligands for peroxisome proliferator-activated receptors *α* and *γ* inhibit chemically induced colitis and formation of aberrant crypt foci in rats. *Cancer Research*.

[53] Yokoyama Y, Xin B, Shigeto T (2007). Clofibric acid, a peroxisome proliferator-activated receptor *α* ligand, inhibits growth of human ovarian cancer. *Molecular Cancer Therapeutics*.

[54] Grabacka M, Plonka PM, Urbanska K, Reiss K (2006). Peroxisome proliferator-activated receptor *α* activation decreases metastatic potential of melanoma cells in vitro via down-regulation of Akt. *Clinical Cancer Research*.

[55] Michalik L, Auwerx J, Berger JP (2006). International union of pharmacology. LXI. Peroxisome proliferator-activated receptors. *Pharmacological Reviews*.

[56] Michalik L, Wahli W (2007). Peroxisome proliferator-activated receptors (PPARs) in skin health, repair and disease. *Biochimica et Biophysica Acta*.

[57] Gupta RA, Dubois RN (2002). Controversy: PPARgamma as a target for treatment of colorectal cancer. *American Journal of Physiology*.

[58] Lefebvre A-M, Chen I, Desreumaux P (1998). Activation of the peroxisome proliferator-activated receptor *γ* promotes the development of colon tumors in C57BL/6J-APC^*M**i**n*^/+ mice. *Nature Medicine*.

[59] Saez E, Tontonoz P, Nelson MC (1998). Activators of the nuclear receptor PPAR*γ* enhance colon polyp formation. *Nature Medicine*.

[60] Girnun GD, Smith WM, Drori S (2002). APC-dependent suppression of colon carcinogenesis by PPAR*γ*. *Proceedings of the National Academy of Sciences of the United States of America*.

[61] Sarraf P, Mueller E, Smith WM (1999). Loss-of-function mutations in PPAR*γ* associated with human colon cancer. *Molecular Cell*.

[62] Tomita S, Kawamata H, Imura J, Omotehara F, Ueda Y, Fujimori T (2002). Frequent polymorphism of peroxisome proliferator activated receptor gamma gene in colorectal cancer containing wild-type K-ras gene. *International Journal of Molecular Medicine*.

[63] Rumi MAK, Ishihara S, Kazumori H, Kadowaki Y, Kinoshita Y (2004). Can PRAR*γ* ligands be used in cancer therapy?. *Current Medicinal Chemistry - Anti-Cancer Agents*.

[64] Mueller E, Smith M, Sarraf P (2000). Effects of ligand activation of peroxisome proliferator-activated receptor *γ* in human prostate cancer. *Proceedings of the National Academy of Sciences of the United States of America*.

[65] Demetri GD, Fletcher CDM, Mueller E (1999). Induction of solid tumor differentiation by the peroxisome proliferator-activated receptor-*γ* ligand troglitazone in patients with liposarcoma. *Proceedings of the National Academy of Sciences of the United States of America*.

[66] Debrock G, Vanhentenrijk V, Sciot R, Debiec-Rychter M, Oyen R, Van Oosterom A (2003). A phase II trial with rosiglitazone in liposarcoma patients. *British Journal of Cancer*.

[67] Varet J, Vincent L, Mirshahi P (2003). Fenofibrate inhibits angiogenesis in vitro and in vivo. *Cellular and Molecular Life Sciences*.

[68] Kasai T, Miyauchi K, Yokoyama T, Aihara K, Daida H (2006). Efficacy of peroxisome proliferative activated receptor (PPAR)-*α* ligands, fenofibrate, on intimal hyperplasia and constrictive remodeling after coronary angioplasty in porcine models. *Atherosclerosis*.

[69] Sato O, Kuriki C, Fukui Y, Motojima K (2002). Dual promoter structure of mouse and human fatty acid translocase/CD36 genes and unique transcriptional activation by peroxisome proliferator-activated receptor *α* and *γ* ligands. *The Journal of Biological Chemistry*.

[70] Jedidi I, Couturier M, Thérond P (2006). Cholesteryl ester hydroperoxides increase macrophage CD36 gene expression via PPAR*α*. *Biochemical and Biophysical Research Communications*.

[71] Huang H, Campbell SC, Bedford DF (2004). Peroxisome proliferator-activated receptor *γ* ligands improve the antitumor efficacy of thrombospondin peptide ABT510. *Molecular Cancer Research*.

[72] Biscetti F, Gaetani E, Flex A (2008). Selective activation of PPAR*α* and PPAR*γ* induces neoangiogenesis through a VEGF-dependent mechanism. *Diabetes*.

[73] Chintalgattu V, Harris GS, Akula SM, Katwa LC (2007). PPAR-*γ* agonists induce the expression of VEGF and its receptors in cultured cardiac myofibroblasts. *Cardiovascular Research*.

[74] Kanata S, Akagi M, Nishimura S (2006). Oxidized LDL binding to LOX-1 upregulates VEGF expression in cultured bovine chondrocytes through activation of PPAR-*γ*. *Biochemical and Biophysical Research Communications*.

[75] Yamakawa K, Hosoi M, Koyama H (2000). Peroxisome proliferator-activated receptor-*γ* agonists increase vascular endothelial growth factor expression in human vascular smooth muscle cells. *Biochemical and Biophysical Research Communications*.

[76] Sarayba MA, Li L, Tungsiripat T (2005). Inhibition of corneal neovascularization by a peroxisome proliferator-activated receptor-*γ* ligand. *Experimental Eye Research*.

[77] Peeters LLH, Vigne J-L, Tee MK, Zhao D, Waite LL, Taylor RN (2006). PPAR*γ* represses VEGF expression in human endometrial cells: implications for uterine angiogenesis. *Angiogenesis*.

[78] Xin B, Yokoyama Y, Shigeto T, Futagami M, Mizunuma H (2007). Inhibitory effect of meloxicam, a selective cyclooxygenase-2 inhibitor, and ciglitazone, a peroxisome proliferator-activated receptor gamma ligand, on the growth of human ovarian cancers. *Cancer*.

[79] Sassa Y, Hata Y, Aiello LP, Taniguchi Y, Kohno K, Ishibashi T (2004). Bifunctional properties of peroxisome proliferator-activated receptor *γ*1 in KDR gene regulation mediated via interaction with both Sp1 and Sp3. *Diabetes*.

[80] Grau R, Punzón C, Fresno M, Iñiguez MA (2006). Peroxisome-proliferator-activated receptor *α* agonists inhibit cyclo-oxygenase 2 and vascular endothelial growth factor transcriptional activation in human colorectal carcinoma cells via inhibition of activator protein-1. *Biochemical Journal*.

[81] Pistrosch F, Herbrig K, Oelschlaegel U (2005). PPAR*γ*-agonist rosiglitazone increases number and migratory activity of cultured endothelial progenitor cells. *Atherosclerosis*.

[82] Wang C-H, Ting M-K, Verma S (2006). Pioglitazone increases the numbers and improves the functional capacity of endothelial progenitor cells in patients with diabetes mellitus. *American Heart Journal*.

[83] Wang C-H, Ciliberti N, Li S-H (2004). Rosiglitazone facilitates angiogenic progenitor cell differentiation toward endothelial lineage: a new paradigm in glitazone pleiotropy. *Circulation*.

[84] Werner C, Kamani CH, Gensch C, Böhm M, Laufs U (2007). The peroxisome proliferator-activated receptor-*γ* agonist pioglitazone increases number and function of endothelial progenitor cells in patients with coronary artery disease and normal glucose tolerance. *Diabetes*.

[85] Wang D, Wang H, Guo Y (2006). Crosstalk between peroxisome proliferator-activated receptor *δ* and VEGF stimulates cancer progression. *Proceedings of the National Academy of Sciences of the United States of America*.

[86] Gensch C, Clever YP, Werner C, Hanhoun M, Böhm M, Laufs U (2007). The PPAR-*γ* agonist pioglitazone increases neoangiogenesis and prevents apoptosis of endothelial progenitor cells. *Atherosclerosis*.

[87] Redondo S, Hristov M, Gümbel D, Tejerina T, Weber C (2007). Biphasic effect of pioglitazone on isolated human endothelial progenitor cells: involvement of peroxisome proliferator-activated receptor-*γ* and transforming growth factor-*β*1. *Thrombosis and Haemostasis*.

[88] Devchand PR, Keller H, Peters JM, Vazquez M, Gonzalez FJ, Wahli W (1996). The PPAR*α*-leukotriene B4 pathway to inflammation control. *Nature*.

[89] Muhlestein JB, May HT, Jensen JR (2006). The reduction of inflammatory biomarkers by statin, fibrate, and combination therapy among diabetic patients with mixed dyslipidemia. The DIACOR (Diabetes and Combined Lipid Therapy Regimen) study. *Journal of the American College of Cardiology*.

[90] Wang T-D, Chen W-J, Lin J-W, Cheng C-C, Chen M-F, Lee Y-T (2003). Efficacy of fenofibrate and simvastatin on endothelial function and inflammatory markers in patients with combined hyperlipidemia: relations with baseline lipid profiles. *Atherosclerosis*.

[91] Cuzzocrea S, Di Paola R, Mazzon E, Genovese T, Muià C, Caputi AP (2004). WY 14643, a potent exogenous PPAR-*α* ligand, reduces intestinal injury associated with splanchnic artery occlusion shock. *Shock*.

[92] Martin-Nizard F, Sahpaz S, Kandoussi A (2004). Natural phenylpropanoids inhibit lipoprotein-induced endothelin-1 secretion by endothelial cells. *Journal of Pharmacy and Pharmacology*.

[93] Staels B, Koenig W, Habib A (1998). Activation of human aortic smooth-muscle cells is inhibited by PPAR*α* but not by PPAR*γ* activators. *Nature*.

[94] Golledge J, Mangan S, Clancy P (2007). Effects of peroxisome proliferator-activated receptor ligands in modulating tissue factor and tissue factor pathway inhibitor in acutely symptomatic carotid atheromas. *Stroke*.

[95] Grabacka M, Reiss K (2008). Anticancer properties of PPAR*α*—effects on cellular metabolism and inflammation. *PPAR Research*.

[96] Delerive P, De Bosscher K, Besnard S (1999). Peroxisome proliferator-activated receptor *α* negatively regulates the vascular inflammatory gene response by negative cross-talk with transcription factors NF-*κ*B and AP-1. *The Journal of Biological Chemistry*.

[97] Ikawa H, Kameda H, Kamitani H (2001). Effect of PPAR activators on cytokine-stimulated cyclooxygenase-2 expression in human colorectal carcinoma cells. *Experimental Cell Research*.

[98] Meade EA, McIntyre TM, Zimmerman GA, Prescott SM (1999). Peroxisome proliferators enhance cyclooxygenase-2 expression in epithelial cells. *The Journal of Biological Chemistry*.

[99] Pasceri V, Chang J, Willerson JT, Yeh ETH (2001). Modulation of C-reactive protein-mediated monocyte chemoattractant protein-1 induction in human endothelial cells by anti-atherosclerosis drugs. *Circulation*.

[100] Marx N, Mach F, Sauty A (2000). Peroxisome proliferator-activated receptor-*γ* activators inhibit IFN-*γ*-induced expression of the T cell-active CXC chemokines IP-10, Mig, and I-TAC in human endothelial cells. *The Journal of Immunology*.

[101] Chen N-G, Han X (2001). Dual function of troglitazone in ICAM-1 gene expression in human vascular endothelium. *Biochemical and Biophysical Research Communications*.

[102] Kliewer SA, Forman BM, Blumberg B (1994). Differential expression and activation of a family of murine peroxisome proliferator-activated receptors. *Proceedings of the National Academy of Sciences of the United States of America*.

[103] Ghosh M, Wang H, Ai Y (2007). COX-2 suppresses tissue factor expression via endocannabinoid-directed PPAR*δ* activation. *Journal of Experimental Medicine*.

[104] Fauconnet S, Lascombe I, Chabannes E (2002). Differential regulation of vascular endothelial growth factor expression by peroxisome proliferator-activated receptors in bladder cancer cells. *The Journal of Biological Chemistry*.

[105] Stephen RL, Gustafsson MCU, Jarvis M (2004). Activation of peroxisome proliferator-activated receptor *δ* stimulates the proliferation of human breast and prostate cancer cell lines. *Cancer Research*.

[106] Hollingshead HE, Killins RL, Borland MG (2007). Peroxisome proliferator-activated receptor-*β*/*δ* (PPAR*β*/*δ*) ligands do not potentiate growth of human cancer cell lines. *Carcinogenesis*.

[107] Gupta RA, Tan J, Krause WF (2000). Prostacyclin-mediated activation of peroxisome proliferator-activated receptor *δ* in colorectal cancer. *Proceedings of the National Academy of Sciences of the United States of America*.

[108] He T-C, Chan TA, Vogelstein B, Kinzler KW (1999). PPAR*δ* is an APC-regulated target of nonsteroidal anti-inflammatory drugs. *Cell*.

[109] Takayama O, Yamamoto H, Damdinsuren B (2006). Expression of PPAR*δ* in multistage carcinogenesis of the colorectum: implications of malignant cancer morphology. *British Journal of Cancer*.

[110] Gupta RA, Wang D, Katkuri S, Wang H, Dey SK, DuBois RN (2004). Activation of nuclear hormone receptor peroxisome proliferator-activated receptor-*δ* accelerates intestinal adenoma growth. *Nature Medicine*.

[111] Singh S, Sadanandam A, Singh RK (2007). Chemokines in tumor angiogenesis and metastasis. *Cancer and Metastasis Reviews*.

[112] Barak Y, Liao D, He W (2002). Effects of peroxisome proliferator-activated receptor *δ* on placentation, adiposity, and colorectal cancer. *Proceedings of the National Academy of Sciences of the United States of America*.

[113] Harman FS, Nicol CJ, Marin HE, Ward JM, Gonzalez FJ, Peters JM (2004). Peroxisome proliferator-activated receptor-*δ* attenuates colon carcinogenesis. *Nature Medicine*.

[114] Reed KR, Sansom OJ, Hayes AJ (2004). PPAR*δ* status and Apc-mediated tumourigenesis in the mouse intestine. *Oncogene*.

[115] Marin HE, Peraza MA, Billin AN (2006). Ligand activation of peroxisome proliferator-activated receptor *β* inhibits colon carcinogenesis. *Cancer Research*.

[116] Wang D, DuBois RN (2008). Peroxisome proliferator-activated receptors and progression of colorectal cancer. *PPAR Research*.

[117] Hatae T, Wada M, Yokoyama C, Shimonishi M, Tanabe T (2001). Prostacyclin-dependent apoptosis mediated by PPAR*δ*. *The Journal of Biological Chemistry*.

[118] Piqueras L, Reynolds AR, Hodivala-Dilke KM (2007). Activation of PPAR*β*/*δ* induces endothelial cell proliferation and angiogenesis. *Arteriosclerosis, Thrombosis, and Vascular Biology*.

[119] Liou J-Y, Lee S, Ghelani D, Matijevic-Aleksic N, Wu KK (2006). Protection of endothelial survival by peroxisome proliferator-activated receptor-*δ* mediated 14-3-3 upregulation. *Arteriosclerosis, Thrombosis, and Vascular Biology*.

[120] Müller-Brüsselbach S, Kömhoff M, Rieck M (2007). Deregulation of tumor angiogenesis and blockade of tumor growth in PPAR*β*-deficient mice. *The EMBO Journal*.

[121] Adamkiewicz J, Kaddatz K, Rieck M, Wilke B, Müller-Brüsselbach S, Müller R (2007). Proteomic profile of mouse fibroblasts with a targeted disruption of the peroxisome proliferator activated receptor-*β*/*δ* gene. *Proteomics*.

[122] Bohman S, Matsumoto T, Suh K (2005). Proteomic analysis of vascular endothelial growth factor-induced endothelial cell differentiation reveals a role for chloride intracellular channel 4 (CLIC4) in tubular morphogenesis. *The Journal of Biological Chemistry*.

[123] Kuppumbatti YS, Rexer B, Nakajo S, Nakaya K, Mira-y-Lopez R (2001). CRBP suppresses breast cancer cell survival and anchorage-independent growth. *Oncogene*.

[124] Abdollahi A, Schwager C, Kleeff J (2007). Transcriptional network governing the angiogenic switch in human pancreatic cancer. *Proceedings of the National Academy of Sciences of the United States of America*.

[125] He T, Lu T, d'Uscio LV, Lam CF, Lee HC, Katusic ZS (2008). Angiogenic function of prostacyclin biosynthesis in human endothelial progenitor cells. *Circulation Research*.

[126] Fan Y, Wang Y, Tang Z (2008). Suppression of pro-inflammatory adhesion molecules by PPAR-*δ* in human vascular endothelial cells. *Arteriosclerosis, Thrombosis, and Vascular Biology*.

[127] Rival Y, Benéteau N, Taillandier T (2002). PPAR*α* and PPAR*δ* activators inhibit cytokine-induced nuclear translocation of NF-*κ*B and expression of VCAM-1 in EAhy926 endothelial cells. *European Journal of Pharmacology*.

[128] Lee C-H, Chawla A, Urbiztondo N, Liao D, Boisvert WA, Evans RM (2003). Transcriptional repression of atherogenic inflammation: modulation by PPAR*δ*. *Science*.

[129] Reichle A, Vogt T, Coras B (2007). Targeted combined anti-inflammatory and angiostatic therapy in advanced melanoma: a randomized phase II trial. *Melanoma Research*.

[130] Vogt T, Hafner C, Bross K (2003). Antiangiogenetic therapy with pioglitazone, rofecoxib, and metronomic trofosfamide in patients with advanced malignant vascular tumors. *Cancer*.

[131] Coras B, Hafner C, Reichle A (2004). Antiangiogenic therapy with pioglitazone, rofecoxib, and trofosfamide in a patient with endemic kaposi sarcoma. *Archives of Dermatology*.

[132] Hau P, Kunz-Schughart L, Bogdahn U (2008). Low-dose chemotherapy in combination with COX-2 inhibitors and PPAR-gamma agonists in recurrent high-grade gliomas—a phase II study. *Oncology*.

[133] McCarty MF, Barroso-Aranda J, Contreras F (2008). PPARgamma agonists can be expected to potentiate the efficacy of metronomic chemotherapy through CD36 up-regulation. *Medical Hypotheses*.

[134] Shearer BG, Steger DJ, Way JM (2008). Identification and characterization of a selective peroxisome proliferator-activated receptor *β*/*δ* (NR1C2) antagonist. *Molecular Endocrinology*.

[135] Kitajima K, Miura S-I, Mastuo Y, Uehara Y, Saku K Newly developed PPAR-*α* agonist (R)-K-13675 inhibits the secretion of inflammatory markers without affecting cell proliferation or tube formation.

